# MicroRNA-9 Enhanced Cisplatin Sensitivity in Nonsmall Cell Lung Cancer Cells by Regulating Eukaryotic Translation Initiation Factor 5A2

**DOI:** 10.1155/2018/1769040

**Published:** 2018-08-05

**Authors:** Qiaoling Pan, Lebo Sun, Dawei Zheng, Ni Li, Huoshun Shi, Jie Song, Guofeng Shao, Guodong Xu

**Affiliations:** Department of Cardiothoracic Surgery, The Affiliated Hospital, Ningbo Medical Center Lihuili Hospital, Ningbo University, Ningbo, Zhejiang 315041, China

## Abstract

We determined the role of microRNA (miR)-9 in regulating cisplatin chemoresistance in nonsmall cell lung cancer (NSCLC) cells. miR-9 and eukaryotic translation initiation factor 5A2 (eIF5A2) levels were examined by reverse transcription–quantitative PCR. Cell Counting Kit-8 and the 5-ethynyl-2′-deoxyuridine (EdU) assay were used to determine the effects of miR-9 mimic or inhibitor on NSCLC cell proliferation and viability, respectively. Bioinformatics was used to analyze the relationship between miR-9 and eIF5A2. Flow cytometry was used to analyze the percentage of apoptotic cells. miR-9 mimic enhanced cisplatin sensitivity, while miR-9 inhibitor produced the opposite result. eIF5A2 was identified as a potential target of miR-9, where miR-9 regulated eIF5A2 expression at mRNA and protein level. miR-9 mimic decreased the expression of eIF5A2 mRNA and protein, while miR-9 inhibitor increased eIF5A2 expression. eIF5A2 knockdown resolved the effects of miR-9 mimic or inhibitor on cisplatin sensitivity. miR-9 may be a potential biomarker for enhancing cisplatin sensitivity by regulating eIF5A2 in NSCLC cells.

## 1. Introduction

Lung cancer is one of the most common malignancies and is a leading cause of cancer-related death worldwide [1]. About 80% of lung cancer diagnosed is nonsmall cell lung cancer (NSCLC) [2]. Cisplatin is the most active chemotherapeutic agent against NSCLC; however, cisplatin resistance often occurs in clinical practice [3]. The process of cisplatin resistance is multifactorial and includes changes in drug accumulation and the apoptosis pathway, drug target interaction, and increased DNA repair [4]. The potential molecular mechanism of the development of cisplatin resistance remains unclear. Therefore, there is a greatly urgent need to identify novel molecules to overcome cisplatin resistance in NSCLC.

MicroRNAs (miRNAs) are small, noncoding, endogenous RNA molecules that play vital roles in gene expression by binding to the 3′ untranslated region (3′UTR) of the target gene mRNA, leading to mRNA degradation or repression of translation [5]. MiRNAs are expressed in some human cancers and play important roles in carcinogenesis [6]. Accumulating evidence has suggested that many differentially regulated miRNAs, such as miR-9, miR-30b, miR-7, and miR-139, are related to many cellular processes, e.g., cell proliferation, invasion and metastasis, differentiation, development, apoptosis, and the processes of drug resistance [7–11]. miR-9 was initially demonstrated to function in neurogenesis and is expressed at different levels in various human cancers. MiR-9 expression is decreased in ovarian cancer, gastric cancer, and oral squamous cell carcinoma, while its expression is increased in other cancers, such as colorectal cancer, breast cancer, and NSCLC [7, 12–16]. MiR-129 and miR-199a-3p overexpression enhance cisplatin sensitivity by targeting P-glycoprotein (P-gp) and downregulating TFAM (transcription factor A, mitochondrial) in human gastric cancer cells and breast cancer cells, respectively [17, 18]. These reports indicate that more in-depth research of miRNAs, which play important roles in cisplatin chemoresistance in NSCLC, is necessary.

In the present study, we investigated the role of miR-9 in cisplatin resistance and explored the mechanism of miR-9 in the expression of genes linked with cisplatin resistance. We found that miR-9 regulates eukaryotic translation initiation factor 5A2 (eIF5A2). Additionally, we confirmed that miR-9 overexpression enhances cisplatin sensitivity in NSCLC cells, while miR-9 inhibitor enhanced NSCLC cell cisplatin resistance. Our study reveals miR-9 as a novel target that may improve NSCLC treatment.

## 2. Materials and Methods

### 2.1. Cell Culture and Reagents

Human NSCLC cell lines (A549, NCI-H358, and NCI-H1299) were obtained from American Type Culture Collection (Manassas, VA, USA) and cultured in RPMI 1640 medium (Gibco, Grand Island, NY, USA) supplemented with 10% fetal bovine serum (FBS; Gibco) and 1% penicillin/streptomycin (Sigma, St. Louis, MO, USA). The cells were maintained at 37°C in a 5% CO_2_ incubator. eIF5A2 small interfering RNA (siRNA) and negative control were purchased from Santa Cruz Biotechnology (Santa Cruz, Dallas, TX, USA). MiR-9 mimic and inhibitor were synthesized by GenePharma (Shanghai, China).

### 2.2. Cell Viability

Cell viability was measured using Cell Counting Kit-8 (CCK-8; Dojindo, Kumamoto, Japan). The NSCLC cells were seeded at a density of 3000 cells per well in 96-well plates with complete medium to grow in a 37°C incubator for 24 h. Then, the culture medium was replaced with medium containing 10% FBS and 0, 0.3125, 0.625, 1.25, 2.5, or 5 *μ*g/mL cisplatin. After a 48-hour incubation, 10 *μ*L CCK-8 solution was added and the cells were incubated for an additional 3 h. The absorbance at 450 nm was detected using an MRX II microplate reader (Dynex Technologies, Chantilly, VA, USA). Relative cell viability was calculated as a percentage of untreated controls.

### 2.3. Transient Transfection with miR-9 Mimic and Inhibitor

The cells were seeded in 6-well plates (2 × 10^5^ cells/well) and transfected with 100 nM miR-9 mimic, 100 nM miR-9 inhibitor, 100 nM eIF5A2 siRNA, or negative siRNA using Lipofectamine 2000 (Invitrogen, Carlsbad, CA, USA) according to the manufacturer's instructions.

### 2.4. Quantitative Reverse Transcription–PCR (RT-qPCR)

Total RNA was extracted from the cells using TRIzol (Invitrogen) according to the manufacturer's instructions. The first-strand complementary DNA (cDNA) was synthesized using a PrimeScript RT Reagent kit (TaKaRa Biotechnology, Dalian, China), followed by PCR amplification in an ABI 7500 Real-Time PCR System (Applied Biosystems, Foster City, CA, USA) and SYBR Green dyestuff (TaKaRa Biotechnology) according to the manufacturers' instructions. Glyceraldehyde-3-phosphate dehydrogenase (GAPDH) and U6 were used as internal controls. miR-9 and eIF5A2 expression were analyzed using the comparative threshold cycle (2^-ΔΔCt^) method [19]. All experiments were repeated at least three times. The target genes were amplified using the following forward and reverse primers: eIF5A2 siRNA, 5′-GCAGACGAAAUUGAUUUCATT-3′, and 5′-UGAAAUCAAUUUCGUCUGCTT-3′;* GAPDH*, 5′-UGACCUCAACUACAUGGUUTT-3′, and 5′-AACCAUGUAGUUGAGGUCATT-3′;* MIR9*, 5′-TCTTTGGTTATCTAGCTGTATGA-3′; miR-9 mimic, 5′-UCUUUGGUUAUCUAGCUGUAUGA-3′ and 5′-AUACAGCUAGAUAACCAAAGAUU-3′; miR-9 inhibitor, 5′-UCAUACAGCUAGAUAACCAAAGA-3′.

### 2.5. Western Blotting

Western blotting was used to determine the protein expression levels in the NSCLC cells. Total protein was extracted using cell lysis buffer (Cell Signaling Technology, Danvers, MA, USA) containing protease inhibitors (Sigma) and quantified using a bicinchoninic acid (BCA) protein assay kit (Beyotime Biotechnology, Shanghai, China). Proteins (40 *μ*g/sample) were separated by 10% sodium dodecyl sulfate–polyacrylamide gel electrophoresis (SDS-PAGE), transferred to polyvinylidene difluoride (PVDF) membranes (Millipore, Billerica, MA, USA), and blocked with 5% defatted milk in Tris-buffered saline and 0.1% Tween 20 (TBS-T) at 37°C for 2 h. After washing three times, the membranes were incubated with primary antibody (eIF5A2, 1:1000 in TBS-T, Santa Cruz Biotechnology) at 4°C overnight. After washing three times, the membranes were incubated with the appropriate horseradish peroxidase–labeled secondary antibody (1:2000) at room temperature for 2 h. The protein bands were detected by chemiluminescence (GE Healthcare, Piscataway, NJ, USA). Data were quantified by the optical density of each band; GAPDH detection on the same membrane was used as the internal control.

### 2.6. 5-Ethynyl-2′-Deoxyuridine (EdU) Staining

Cell proliferation was examined using a Click-iT EdU Imaging Kit (Invitrogen) according to the manufacturer's instructions.

### 2.7. Flow Cytometry

NSCLC cells were transfected with miR-9 mimic, inhibitor, or negative control for 48 h and incubated for 48 h with cisplatin as follows: A549 cells, 1.735 *μ*g/mL; NCI-H358 cells, 3.027 *μ*g/mL; NCI-H1299 cells, 4.950 *μ*g/mL. Then, 2 × 10^5^ cells were collected and washed twice with phosphate-buffered saline (PBS). Apoptotic cells were detected with annexin V–fluorescein isothiocyanate/propidium iodide (FITC/PI) using an Annexin V-FITC Cell Apoptosis Detection Kit (BD Biosciences, Franklin Lakes, NJ, USA) according to the kit's protocol.

### 2.8. Statistical Analysis

The data are reported as the means ± standard deviation (SD) and were analyzed using GraphPad Prism 5 (GraphPad Software Inc., La Jolla, CA, USA). All experiments were repeated at least three times. Groups were compared using Student's* t*-test or one-way analysis of variance (ANOVA).* P *< 0.05 was considered to indicate statistically significant difference.

## 3. Results

### 3.1. MiR-9 Expression Correlates Positively with NSCLC Cell Cisplatin Sensitivity

**Figure 1:**
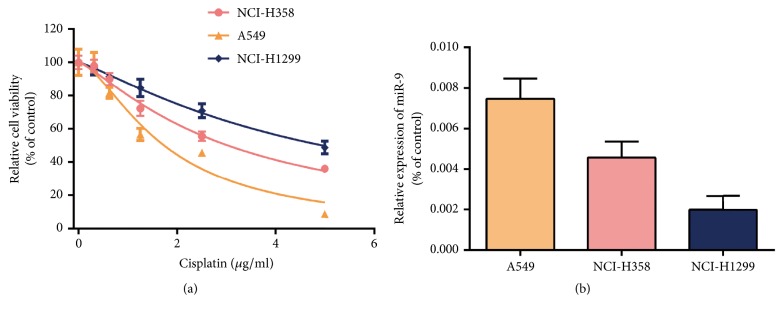
**Cisplatin sensitivity in NSCLC cells**. (a) CCK-8 determination of cell viability after treatment with 0, 0.3125, 0.625, 1.25, 2.5, or 5 *μ*g/mL cisplatin. (b) RT-qPCR detection of miR-9 expression in cells. U6 was used as an internal control.

To research the relationship between cisplatin sensitivity and miR-9 expression in NSCLC cells, CCK-8 was used to examine cell viability following a 48-hour treatment with 0, 0.3125, 0.625, 1.25, 2.5, or 5 *μ*g/mL cisplatin. A549 cells were the most sensitive to cisplatin among the three NSCLC cell lines (median inhibitory concentration [IC50]: A549, 1.735 *μ*g/mL; NCI-H358, 3.027 *μ*g/mL; NCI-H1299; 4.950 *μ*g/mL)** (Figure 1(a))**. Interestingly, A549 cells also had the highest miR-9 expression among the three cell lines** (Figure 1(b))**. These results indicate that miR-9 expression correlates positively with cisplatin sensitivity in NSCLC cells.

### 3.2. MiR-9 Regulates NSCLC Cell Cisplatin Sensitivity and Apoptosis

**Figure 2:**
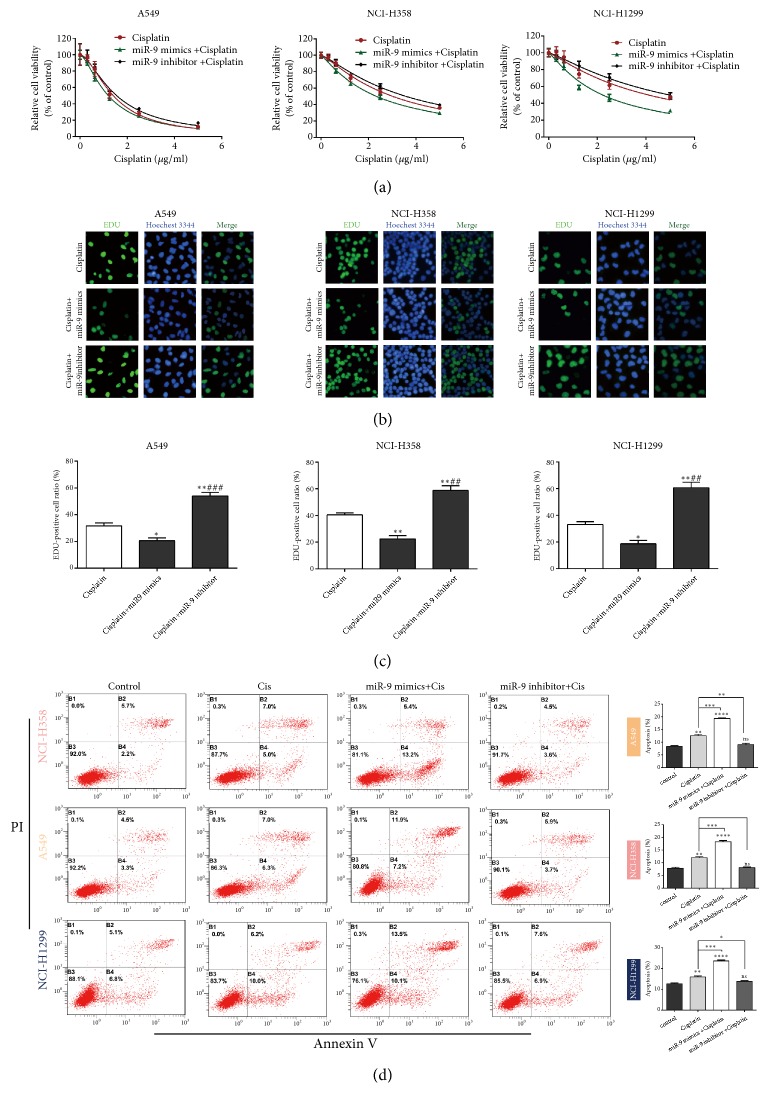
**MiR-9 overexpression enhances NSCLC cell sensitivity to cisplatin.** (a) CCK-8 examination of cell viability following treatment with cisplatin alone or with cisplatin plus miR-9 mimic or inhibitor. (b, c) EdU staining analysis of cell proliferation following treatment with cisplatin alone or with cisplatin plus miR-9 mimic or inhibitor. *∗P* < 0.05, *∗∗P* < 0.01 vs. cisplatin; ##*P* < 0.01 vs. cisplatin+miR-9 mimic. (d) Flow cytometry determination of the percentage of apoptotic cells following treatment with cisplatin alone or with cisplatin plus miR-9 mimic or inhibitor. *∗P* < 0.05,*∗∗P* < 0.01, and *∗∗∗P* < 0.001.

To determine whether miR-9 is related to cisplatin resistance in NSCLC cells, NSCLC cells were transfected with cisplatin alone or with cisplatin plus miR-9 mimic or inhibitor for 48 h, and then cell viability was detected using CCK-8.  Figure 2(a) shows that compared with cisplatin alone, miR-9 mimic enhanced cisplatin sensitivity, while the miR-9 inhibitor decreased the effect of cisplatin in the cells. The EdU assay also proved that compared with cisplatin alone, miR-9 mimic could decrease the cell proliferation rate, while the miR-9 inhibitor had the opposite effect (Figures 2(b) and 2(c)). Flow cytometry showed that miR-9 mimic or miR-9 inhibitor increased the apoptosis rate compared with cisplatin alone (Figure 2(d)).

### 3.3. EIF5A2 Is a Direct Target Gene of miR-9 in NSCLC Cells

**Figure 3:**
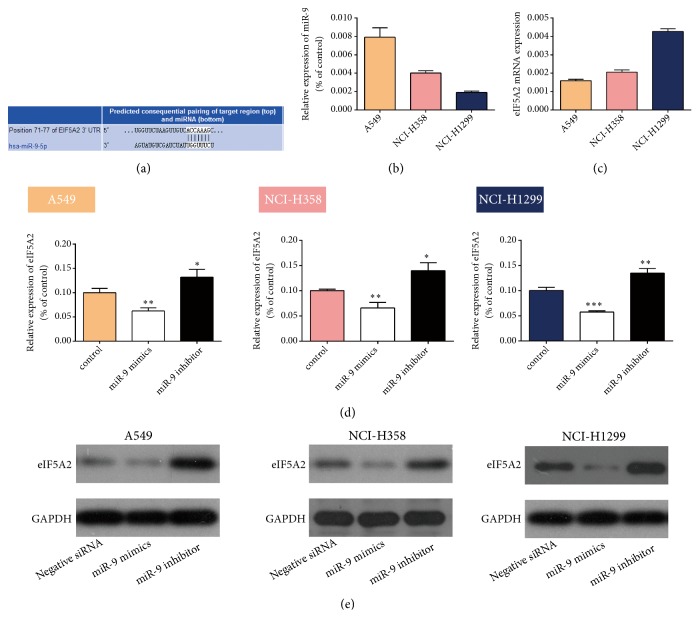
***EIF5A2* is a direct target of miR-9 in NSCLC cells.** (a) TargetScan prediction of a miR-9 binding site in the* EIF5A2* 3′UTR. (b) RT-qPCR analysis shows that miR-9 expression was the highest in the A549 cells. (c) RT-qPCR measurement of eIF5A2 expression. (d) RT-qPCR detection of eIF5A2 levels following transfection with miR-9 mimic or inhibitor. *∗P* < 0.05, *∗∗P* < 0.01, and *∗∗∗P* < 0.001 vs. control. (e) Western blot shows increased eIF5A2 levels following transfection with miR-9 mimic and decreased eIF5A2 levels following transfection with miR-9 inhibitor as compared with negative siRNA.

To further demonstrate whether miR-9 is involved in eIF5A2 expression, we used TargetScan (www.targetscan.org) to predict the relationship between miR-9 and eIF5A2 and found that* EIF5A2* is a target gene of miR-9 (Figure 3(a)). RT-qPCR measurement of* MIR9* and* EIF5A2* mRNA expression showed that the A549 cells had the highest miR-9 expression but the lowest eIF5A2 expression among the NSCLC cell lines (Figures 3(b) and 3(c)), indicating that miR-9 correlates negatively with eIF5A2 expression. RT-qPCR and western blotting confirmed that compared with the negative control, miR-9 mimic could increase eIF5A2 mRNA and protein expression and that miR-9 inhibitor could decrease expression (Figures 3(d) and 3(e)).

### 3.4. EIF5A2 Knockdown Enhances NSCLC Cell Cisplatin Sensitivity

**Figure 4:**
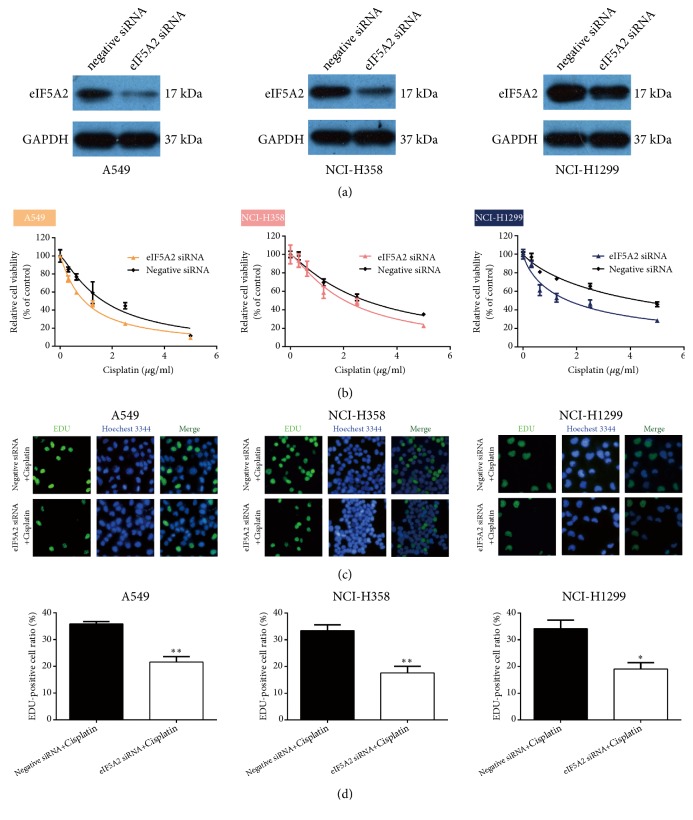
**EIF5A2 knockdown enhances NSCLC cell sensitivity to cisplatin. **(a) Western blot shows decreased eIF5A2 levels following transfection with eIF5A2 siRNA as compared with negative siRNA. (b) CCK-8 showing decreased cell viability following transfection with eIF5A2 siRNA as compared to negative siRNA. (c) EdU staining determination of cell proliferation following treatment with cisplatin with and without eIF5A2 siRNA transfection. (d) Histogram representation of the EdU-positive cells. *∗P*< 0.05 and *∗∗P* < 0.01 vs. negative siRNA.

We used CCK-8 to detect cell viability following treatment with cisplatin with or without eIF5A2 siRNA and found that eIF5A2 knockdown significantly increased cisplatin sensitivity in the NSCLC cells (Figure 4(b)). The EdU assay was used to determine cell proliferation and showed that compared with the cisplatin alone group, cisplatin treatment plus eIF5A2 siRNA transfection reduced cell proliferation (Figure 4(c)). Western blotting demonstrated the interference efficiency of eIF5A2 (Figure 4(a)). These results demonstrate that inhibiting eIF5A2 significantly increases the cisplatin sensitivity of NSCLC cells.

### 3.5. MiR-9 Enhances NSCLC Cell Sensitivity to Cisplatin by Inhibiting eIF5A2 Expression

**Figure 5:**
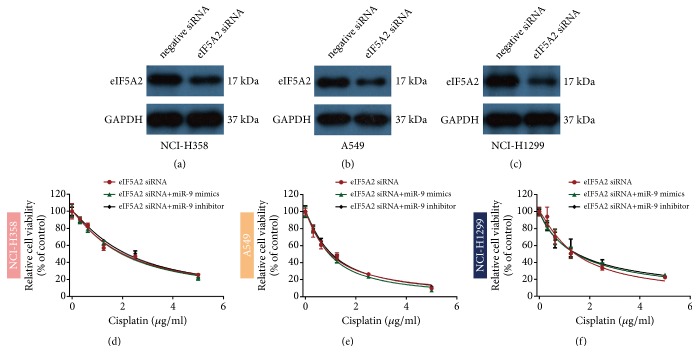
**MiR-9 enhances NSCLC cell sensitivity to cisplatin by inhibiting eIF5A2 expression.** (a–c) Western blot showing eIF5A2 expression after siRNA transfection. (d–f) CCK-8 measurement of cisplatin sensitivity following siRNA transfection with miR-9 mimic or inhibitor.

To verify whether miR-9 enhances NSCLC cell cisplatin sensitivity by regulating eIF5A2 expression, we silenced eIF5A2 and transfected the cells with miR-9 mimic or inhibitor before examining the cell viability via CCK-8. There was no significant difference among the three groups (Figures 5(d) and 5(f)). Western blotting detected the interference efficiency of eIF5A2 (Figures 5(a)–5(c)). These findings prove that miR-9 may increase NSCLC cell sensitivity to cisplatin by inhibiting eIF5A2 expression.

## 4. Discussion

Lung cancer is a major cause of cancer-related death worldwide. As a chemotherapy drug, cisplatin is used for extensive treatment in NSCLC, although cisplatin resistance presents a significant barrier to the improvement of the long-term outcome of patients with NSCLC [20, 21]. Many factors enhance the sensitivity of NSCLC cells to cisplatin and may reveal novel targets for therapy. Therefore, it is very important to identify therapeutic targets to improve the cisplatin sensitivity of NSCLC cells.

MiRNAs play a crucial role in many biological processes, such as proliferation, apoptosis, and differentiation, by modulating gene expression at posttranscriptional level [22]. Aberrant miRNAs can affect the expression of target genes, which may modulate cell death signaling pathways, drug target proteins, and cell cycle–related proteins. This may lead to cancer cell resistance to various therapies [23]. Numerous researchers have demonstrated that miRNAs could be a novel tool for cancer diagnostics and therapeutics [24]. For example, miR-1271 upregulation enhances cisplatin sensitivity in gastric cancer cells and miR-106b-5p upregulation or PKD2 (polycystin 2, transient receptor potential cation channel) inhibition could increase NSCLC cell sensitivity to cisplatin [24, 25]. In addition, recent studies have suggested that miR-9 is closely related to cell metastasis, drug resistance, cell proliferation, and apoptosis [15, 16, 26]. However, the effect of miR-9 on cisplatin resistance in NSCLC cells remains unclear. In the present study, we explored the effect of miR-9 mimic or inhibitor on the cisplatin resistance of NSCLC cells. miR-9 mimic transfection was followed by miR-9 overexpression, which enhanced NSCLC cell sensitivity to cisplatin, while inhibiting miR-9 enhanced cisplatin resistance in the cells. The EdU assay and flow cytometry showed that miR-9 increased apoptotic cells and inhibited cell proliferation, all of which may be mediated by the inhibition of eIF5A2, indicating that miR-9 may function as a tumor oncogene in NSCLC cells.

EIF5A2, an isoform of eIF5A and located on chromosome 3q26, was first discovered in ovarian carcinoma and has been identified as a novel oncogene in many human cancer cells [27]. eIF5A2 plays critical roles in cell proliferation, invasion and metastasis, and apoptosis. Previous studies have confirmed that eIF5A2 is upregulated in various cancer types, such as gastric cancer, bladder cancer, hepatocellular carcinoma (HCC), and colon cancer [28–32]. Silencing eIF5A2 enhanced the therapeutic efficacy of drugs in HCC, colorectal cancer, and breast cancer [30, 33, 34]. Furthermore, it has been shown that knockdown of eIF5A2 enhances NSCLC sensitivity to cisplatin by preventing epithelial–mesenchymal transition (EMT), reducing the migration and invasive capabilities of NSCLC cells [35]. In the present study, the eIF5A2 knockdown group had greater cisplatin sensitivity than the negative siRNA group. Moreover, miR-9 regulated eIF5A2 expression, where miR-9 mimic and inhibitor downregulated and upregulated eIF5A2, respectively, at protein and mRNA level.

Increasing evidence has proved that miRNAs regulate target gene expression negatively by promoting translational repression, mRNA cleavage, or a combination of both, which occurs mostly through direct targeting of the 3′UTRs of mRNAs [17, 36]. In the present study, bioinformatics analysis confirmed that eIF5A2 is a functional target of miR-9 in NSCLC cells. miR-9 mimic transfection decreased eIF5A2 expression, but transfection with miR-9 inhibitor enhanced eIF5A2 expression, suggesting that* EIF5A2* is a target gene of miR-9 in NSCLC cells. We then detected miR-9 expression in three NSCLC cell lines and found that miR-9 expression was the highest in A549 cells, which were also the most sensitive to cisplatin among the three cell lines. Furthermore, miR-9 levels correlated negatively with eIF5A2 expression. Silencing eIF5A2 revealed that there was no significant difference in cisplatin sensitivity with or without miR-9 mimic and inhibitor, suggesting that miR-9 regulates cisplatin sensitivity in NSCLC cells by downregulating eIF5A2.

In conclusion, this study demonstrates that miR-9 expression correlates with cisplatin sensitivity in NSCLC cells. miR-9 overexpression decreases eIF5A2 and increases cisplatin sensitivity, but inhibiting miR-9 decreases cisplatin sensitivity. Taken together, these data indicate that miR-9 could be an important therapeutic biomarker for enhancing chemosensitivity to cisplatin treatment.

## Data Availability

The data used to support the findings of this study are available from the corresponding author upon request.
